# Nursery Ethics

**Published:** 1895-08

**Authors:** 


### "NURSERY ETHICS"

Is the title of a valuable little volume upon parental government which The Merriam Company are about to issue. It is from the pen of Mrs. Florence Hull Winterburn, and is marked by the practical good sense and deep insight into human nature which have distinguished the many short articles that have appeared from time to time under her name. It is in press, to appear early in September. It is said to be the most striking and original volume which has yet been published upon a topic that at the present time engrosses so large a share of public attention. The author's excellent work in the magazine *Childhood* has established her reputation as an authority upon child training, and the appearance of this book is awaited with much interest.

